# Increased Risk for Invasive Breast Cancer Associated with Hormonal Therapy: A Nation-Wide Random Sample of 65,723 Women Followed from 1997 to 2008

**DOI:** 10.1371/journal.pone.0025183

**Published:** 2011-10-06

**Authors:** Jung-Nien Lai, Chien-Tung Wu, Pau-Chung Chen, Chiun-Sheng Huang, Song-Nan Chow, Jung-Der Wang

**Affiliations:** 1 Institute of Traditional Medicine, School of Medicine, National Yang-Ming University, Taipei, Taiwan; 2 Department of Obstetrics and Gynecology, Yangming Branch, Taipei City Hospital, Taipei, Taiwan; 3 Department of Chinese Medicine, Taipei City Hospital, Taipei, Taiwan; 4 Institute of Occupational Medicine and Industrial Hygiene, College of Public Health, National Taiwan University, Taipei, Taiwan; 5 Department of Surgery, College of Medicine, National Taiwan University, Taipei, Taiwan; 6 Department of Surgery, National Taiwan University Hospital, Taipei, Taiwan; 7 Department of Obstetrics & Gynecology, National Taiwan University Hospital, Taipei, Taiwan; 8 Department of Public Health, College of Medicine, National Cheng Kung University, Tainan, Taiwan; 9 Department of Occupational and Environmental Medicine, National Cheng Kung University Hospital, Tainan, Taiwan; National Taiwan University Hospital, Taiwan

## Abstract

**Background:**

Hormonal therapy (HT) either estrogen alone (E-alone) or estrogen plus progesterone (E+P) appears to increase the risk for breast cancer in Western countries. However, limited information is available on the association between HT and breast cancer in Asian women characterized mainly by dietary phytoestrogens intake and low prevalence of contraceptive pills prescription.

**Methodology:**

A total of 65,723 women (20–79 years of age) without cancer or the use of Chinese herbal products were recruited from a nation-wide one-million representative sample of the National Health Insurance of Taiwan and followed from 1997 to 2008. Seven hundred and eighty incidents of invasive breast cancer were diagnosed. Using a reference group that comprised 40,052 women who had never received a hormone prescription, Cox proportional hazard models were constructed to determine the hazard ratios for receiving different types of HT and the occurrence of breast cancer.

**Conclusions:**

5,156 (20%) women ever used E+P, 2,798 (10.8%) ever used E-alone, and 17,717 (69%) ever used other preparation types. The Cox model revealed adjusted hazard ratios (HRs) of 2.05 (95% CI 1.37–3.07) for current users of E-alone and 8.65 (95% CI 5.45–13.70) for current users of E+P. Using women who had ceased to take hormonal medication for 6 years or more as the reference group, the adjusted HRs were significantly elevated and greater than current users and women who had discontinued hormonal medication for less than 6 years. Current users of either E-alone or E+P have an increased risk for invasive breast cancer in Taiwan, and precautions should be taken when such agents are prescribed.

## Introduction

Many studies have shown that hormonal therapy (HT) with estrogen plus progesterone (E+P) increases the risk for breast cancer [1]–[5]. In particular, the results of the observational Million Women Study (MWS) [2] and the Nurses' Health Study (NHS) [6] suggest that there is an association between estrogen and breast cancer in routine gynaecology practices [7]. By contrast, the Women's Health Initiative (WHI) [1] did not detect a higher level of risk in the estrogen alone (E-alone) group. Unfortunately, long-term randomised controlled trials that are designed to assess the above inconsistent findings appear to be difficult due to ethical, economical, and practical considerations [7]. Besides, most studies [1]–[6], [8], have not deliberately controlled the factor of herbs consumption [9], which may contain estrogen-like substances [10]–[14] and are increasingly widely used in women [15], [16]. All the above factors leave lots of space for more studies. Large-scale retrospective data can help to assess the association between breast cancer and HT in real practice. We excluded women ever prescribed traditional Chinese medicines and compared hazard ratios for breast cancer across categories of different types of HT in a large retrospective Taiwan cohort.

## Results

The database contained a total of 2,461 prevalent cases of invasive breast cancer that were diagnosed between 1997 and 2008. Within this population, we identified 780 patients who were newly diagnosed with invasive breast cancer during the ten-year study period (1999–2008) and were between the ages of 20 and 79 years. Table 1 summarises the characteristics of the study population, the number of incidents of invasive breast cancer, and the proportion of ever users of HT at baseline according to the patient age. The proportion of different types of HT varied substantially according to different age groups: E-alone HT was used mainly by women aged 40 to 59 years, E+P HT mainly by women aged 30 to 49 years, and other types of HT by women aged 20 to 39 years. The majority of E-alone HT use comprised conjugated equine estrogen, and 0.625 mg/day was the dose that was most commonly prescribed. Combination therapy was frequently prescribed in a sequential manner that usually constituted a combination of 0.625 mg conjugated equine estrogen, and medroxyprogesterone acetate comprised the great majority of all progesterone use. The average prescribed cumulated estrogen doses of HT for the E-alone HT, the mixed regimen (E-alone and E+P), and the E+P HT group were 281.0, 297.3, and 170.8 mg, respectively. The average age of the women at the time of recruitment was 40.5±15.2 years.

**Table 1:** Baseline demographic and clinical characteristics of the Taiwanese cohort (n = 65,723) stratified by different types of hormone replacement therapy followed from 1997 to 2008.

Characteristic	Never users	E-alone	E+P	E-alone and E+P	P-alone
Total No.	40,052	5,156	2,798	9,961	7,756
New breast cancer, No. (%)	497(1.2)	69(1.3)	41(1.5)	106(1.1)	67(0.9)
Incidence rate+	103.4	111.5	122.1	88.7	72.0
Mean (SD) age at inclusion, years	41.9(16.1)	48.6(13.1)	44.0(14.4)	37.8(12.2)	30.7(8.3)
Age groups at inclusion, years No. (%)					
20–29	11,171(27.9)	434(8.4)	548(19.6)	3,033(30.5)	3,970(51.2)
30–39	9,931(24.8)	732(14.2)	618(22.1)	2,714(27.3)	2,748(35.4)
40–49	7,049(17.6)	1,741(33.8)	621(22.2)	2,614(26.2)	889(11.5)
50–59	4,398(11.0)	1,164(22.6)	552(19.7)	1,011(10.2)	59(0.8)
60–69	4,556(11.4)	717(13.9)	344(12.3)	449(4.5)	54(0.7)
70–79	2,947(7.4)	375(7.3)	119(4.3)	140(1.4)	36(0.5)
Cumulative estrogen dose, mean (SD), DDD	0	281.0(714.7)	170.8(427.3)	297.3(686.3)	0
Cumulative progesterone dose, mean (SD), DDD	0	0	139.5(407.1)	148.4(545.1)	128.9(7177.0)

*E+P, estrogen-progesterone combination; E-alone, estrogen-alone; P-alone, progesterone only; E-alone and E+P ( the mixed regimen), combinations of the above types (E+P, E-alone); DDD, defined daily dose.

+ Average annual per 100,000.

In comparison to women aged 30 to 39 years, the hazard ratios (HRs) for invasive breast cancer among never users of HT were 1.88 (95% CI 1.49–2.37) for women aged 40 to 49 years and 1.73 (95% CI 1.32–2.26) for women aged 50 to 59 years. The HR calculated from the Cox regression model for ever users of HT was significantly higher than that determined for never users. Table 2 summarises the different magnitudes of invasive breast cancer risks for the different types of HT (E-alone, E+P, E-alone and E+P combination, and progesterone-alone) after adjusting for age. The adjusted HR for the development of invasive breast cancer was significantly increased by 2.03-fold (95% CI 1.36–3.05) for current users of E-alone HT, by 2.67-fold (95% CI 1.75–4.06) for current users of mixed regimen, but it was even higher for E+P (HR 8.72, 95% CI 5.50–13.82). The magnitudes of HR were higher if we limited the analysis to postmenopausal women (55–79 years of age). Such effects decreased markedly if the women no longer received medications containing estrogens. In general, the HR dropped to a risk near baseline when the medications had not been used for more than 5 years.

**Table 2:** Number (No.) of new cases, population-at-risk, and estimated hazard ratios (HR), 95% confidence intervals (CI) based on multivariate Cox regression model on a random sample of the National Health Insurance Research Database followed from 1997 to 2008 stratified by age in 1997.

	Women aged 20 to 79 years		Women aged 55 to 79 years	
HRT use at baseline	No. Cases/population	HR(95% CI)	No. Cases/population	HR(95% CI)
**Never users (referents)**	497/40,052	1	124/9,939	1
**Estrogen-alone**				
Current users	25/656	2.03(1.36–3.04)	6/135	3.13(1.37–7.12)
Last use 1–3 years previously	18/815	1.22(0.76–1.96)	7/199	2.58(1.20–5.53)
Last use 4–5 years previously	11/832	0.73(0.40–1.33)	4/242	1.21(0.45–3.27)
Last use > = 6 years previously	15/2,853	0.29(0.17–0.49)	6/1,072	0.43(0.19–0.97)
**Estrogen-progesterone combination**				
Current users	19/196	8.74(5.52–13.86)	11/23	51.64(27.74–96.11)
Last use 1–3 years previously	7/329	2.16(1.02–4.55)	5/26	16.95(6.91–41.58)
Last use 4–5 years previously	6/394	1.35(0.60–3.03)	2/32	4.54(1.12–18.38)
Last use > = 6 years previously	9/1,879	0.27(0.14–0.53)	3/642	0.34(0.11–1.06)
**Others**				
**Estrogen-alone** and **Estrogen-progesterone combination**				
Current users	51/2,065	2.01(1.50–2.68)	4/133	2.00(0.73–5.43)
Last use 1–3 years previously	23/2,215	0.92(0.61–1.40)	2/119	1.17(0.29–4.74)
Last use 4–5 years previously	17/1,895	0.68(0.42–1.10)	2/156	0.90(0.22–3.65)
Last use > = 6 years previously	15/3,786	0.26(0.16–0.44)	1/584	0.12(0.02–0.88)
**Progesterone-alone**				
Current users	14/766	2.78(1.63–4.77)	0/13	-
Last use 1–3 years previously	15/1,311	1.71(1.02–2.88)	0/26	-
Last use 4–5 years previously	12/1,421	1.19(0.67–2.13)	0/25	-
Last use > = 6 years previously	26/4,258	0.70(0.47–1.04)	0/47	-

*HRT, hormone replacement therapy; Other types, including mixed combinations of the above types (estrogen-progesterone combination, estrogen-alone) and progesterone only.

When the reference group comprised women who ceased the intake of hormonal medication for 6 years or more, the adjusted HRs were still significantly elevated and demonstrated greater magnitudes than current users and women who ceased the intake of hormonal medication for less than 6 years, and the effect of age was maintained (Table 3). In particular, the risk for invasive breast cancer in E+P was also higher than that in E-alone. When we limited the analysis to current users (namely those who were prescribed HT within one year before the diagnosis of breast cancer or at the end of 2008), a statistically significant (*P*<.001) linear dose–response relationship was observed between the risk for the development of invasive breast cancer and the prescribed dose of E-alone, mixed regimen and E+P HT. The comparison of current HT users to women who had ceased to use HT at least five years prior revealed that an increase of every 30 defined daily dose of E-alone, mixed regimen or E+P HT was significantly associated with an increased hazard ratio for invasive breast cancer among current HT users.

**Table 3:** Number (No.) of new cases, population-at-risk, and estimated hazard ratios (HR), 95% confidence intervals (CI) estimated from multivariate Cox regression model on a random sample of the National Health Insurance Research Database among women with hormone replacement therapy stratified by age and followed from 1999 to 2008.

	Women aged 20 to 79 years		Women aged 55 to 79 years	
HRT use at baseline	No. Cases/population	HR(95% CI)	No. Cases/population	HR(95% CI)
**Estrogen-alone**				
Last use > = 6 years previously	15/2,853	1.00	6/1,072	1.00
Last use 4–5 years previously	11/832	2.65(1.21–5.79)	4/242	2.93(0.82–10.40)
Last use 1–3 years previously	18/815	4.47(2.24–8.92)	7/199	5.94(1.99–17.68)
Current users	25/656	7.73(4.02–14.84)	6/135	7.74(2.47–24.23)
Per 30 DDD	-	1.07(1.04–1.09)	-	1.16(1.05–1.28)
**Estrogen-progesterone combination**				
Last use > = 6 years previously	9/1,879	1.00	3/642	1
Last use 4–5 years previously	6/394	9.69(3.40–27.67)	2/32	13.85(2.31–83.00)
Last use 1–3 years previously	7/329	14.50(5.26–40.01)	5/26	43.52(10.20–185.79)
Current users	19/196	61.89(27.37–139.96)	11/23	143.90(39.90–518.96)
Per 30 DDD	-	1.40(1.29–1.51)	-	1.71(1.50–1.95)
**Others**				
**Estrogen-alone** and **Estrogen-progesterone combination**				
Last use > = 6 years previously	15/3,786	1	1/584	1
Last use 4–5 years previously	17/1,895	2.71(1.35–5.44)	2/156	7.09(0.64–78.22)
Last use 1–3 years previously	23/2,215	3.80(1.97–7.34)	2/119	9.51(0.86–105.20)
Current users	51/2,065	8.18(4.58–14.62)	4/133	14.58(1.62–131.26)
Per 30 DDD	-	1.09(1.05–1.12)	-	1.12(1.06–1.19)
**Progesterone-alone**				
Last use > = 6 years previously	26/4,258	1	0/47	-
Last use 4–5 years previously	12/1,421	1.82(0.92–3.63)	0/25	-
Last use 1–3 years previously	15/1,311	2.59(1.36–4.94)	0/26	-
Current users	14/766	4.32(2.22–8.38)	0/13	-
Per 30 DDD	-	-	-	-

*HRT, hormone replacement therapy; Other types, including mixed combinations of the above types (estrogen-progesterone combination, estrogen-alone) and progesterone only.

With last use of hormones more than 6 years ago as the referents, we tested different periods of last use and the potential dose-response relationship with an increment of 30 DDD (defined daily dose) among current users.

## Discussion

To our knowledge, this is the first study to employ a nation-wide representative cohort to examine the increased risk for invasive breast cancer among women in Taiwan who are undergoing treatment with HT. Because this issue has been heavily debated internationally, we must be cautious about potential confounding factors prior to generating any inferences. However, the following arguments provide a warning to individuals concerning the possible risks of HT. First, because the NHIRD collects all prescription information prospectively, we can rule out the possibility of a recall bias concerning intake dosages and different types of prescriptions: E-alone, E+P HT, progesterone only, or a mixed regimen. Second, in the present study, we included all of the patients who were newly diagnosed with invasive breast cancer between 1999 and 2008 from a simple random sample of one million subjects among the insured general population. Because the rate of insured individuals has been consistently above 96% since 1997, we can rule out the possibility of a selection bias. In fact, our current estimate of 63.7 new breast cancer cases per million person-years is very close to the 66.2 cases per million person-years that was calculated from the National Cancer Registry of Taiwan in 2005. Third, to minimise potential confounding factors according to the indication, we limited the analysis to postmenopausal women (55–79 years old of age), and an association between HT and invasive breast cancer risk was still observed, as summarised in Table 2. Indeed, this direct effect of age on breast cancer risk corroborates the result obtained in previous studies [17], [18]. Fourth, we attempted to control for potential confounding factors associated with the indication by evaluating ex-users who had ceased hormone medication intake for 6 years or more as the reference group. Table 3 shows that a discontinued exposure to HT significantly decreases the risk for invasive breast cancer among women of both reproductive age and postmenopausal age, and there is a consistent trend that demonstrates a decreased risk that is accompanied by a longer period of discontinued exposure. Moreover, a significant linear dose-response relationship was observed for an increment of 30 defined daily doses among current users in comparison with ex-users who had ceased HT for more than 6 years. Because we minimized the risk for data falsification, we tentatively concluded that in Taiwan, current users of E-alone and/or E+P HT might have an increased risk for invasive breast cancer.

The present findings shown in Tables 2 and 3 corroborate the results of the WHI, which demonstrated that estrogen plus progesterone might be associated with an increased risk for breast cancer. Although this study was not a randomised control trial, it included a cohort of one million random samples that has been shown to be representative of the population in Taiwan. Since the WHI published their major findings in 2002, Taiwanese women have demonstrated a period of reduced prescription hormone intake [19]. In fact, due to fear of any challenge from the patient and/or her family, physicians typically do not prescribe hormones to any patient if there is any doubt regarding the possible beneficial or potential harmful effects of hormones [20], [21]. Moreover, the National Health Insurance in Taiwan stipulates a three-month upper limit for repeat prescriptions, which provides another constraint on any unnecessary or harmful medications. The frequency of HT prescribing in Taiwan was somewhat expected lower than in the United States due to this HT prescribing policy (Table S1). As shown in Tables 2 and 3, a positive association between E+P and the occurrence of invasive breast cancer in women in Taiwan was still observed under the above circumstances, we think that such a positive association is real.

Breast cancer incidence was notably high in Western countries and the rates of all age groups exceeded those from Taiwan, Japan, and Hong Kong (Table S2). Although the WHI and some studies in Western countries [22]–[24] did not observe a positive association between breast cancer and an estrogen exposure of less than 5 years, there have been few reports in Asia to test this hypothesis. As Asian women are generally slimmer than women from Western countries, they might be exposed to a relatively high dose of estrogen based on the guideline recommendations. Given the 12 years of follow-up that were available for testing this hypothesis, we were more likely to detect a positive effect between the prescription of E-alone and invasive breast cancer. Under the NHI system in Taiwan, all individuals who receive HT are required to undergo a follow-up by their physicians no longer than three months. These guidelines increase the likelihood of breast cancer detection during an earlier stage of disease rather than during the invasive stage [25]. The positive association persisted under the following different settings. When we limited our analysis to postmenopausal women, or when women who have ceased to use E-alone for more than 6 years were used as the reference group, a positive linear dose-response relationship was observed among current users (Table 3). Considering evidence obtained in observational studies that were conducted in Western countries, we suspect that this association is real with respect to the prescribed doses administered in women in Taiwan. As the prevalence of HT exposure differs between Taiwan and other countries, however, we would also expect the burden of HT attributable cancer incidence to differ across the countries, which is of potential interest for public health policy makers. This observation warrants further studies to determine whether a differential genetic susceptibility between Asian and Caucasian postmenopausal women and the use of universal precautions.

In this study and others [26], the combined estrogen-progesterone regimen appeared to be a maximally effective type of estrogen in regard to the occurrence of breast cancer beyond which other types of HT regimens had no or less effect. Adding a progesterone to an estrogen therapy was associated with greater increases in breast cancer risk and with longer washout period for reducing the residual effects compared with the use of estrogen alone. Just as importantly, the disparity between the associations became even larger when we limited our analysis to postmenopausal women. We hypothesize that progesterone play an important role on the occurrence of breast cancer especially in whom has relatively lower endogenous estrogen levels. And, further analysis found that women use mixed regimen were no more susceptible to breast cancer than those use E-alone HT either in reproductive or in postmenopausal age group. The evidence we obtained indicated that the particular type of HT, adding a progesterone continuously to a postmenopausal hormone program, will be more likely to increase the occurrence of breast cancer. However, before drawing any conclusions from the data, further studies are warranted to be replicated with a larger sample size.

The present study has some limitations. First, because the identities of the patients were not available in the NHI reimbursement database, we were unable to obtain any histopathology reports to verify the diagnoses. However, because approval for the registration of invasive breast cancer as a catastrophic illness is based on pathology and/or cytologic evidence and is followed by a full waiver of copayment, such a diagnosis is made only after very serious considerations and is generally accurate. The diagnostic accuracy of invasive breast cancer among the NHI data is corroborated by the considerable agreement between the incidence rate calculated herein and that determined by the National Cancer Registry of Taiwan, in which 95% of the breast cancers are accompanied by histopathologic validation. Second, we were unable to contact the patients directly regarding their use of HRT due to the encryption of their identification numbers; therefore, we cannot exclude the possibility that the subjects used additional phytoestrogenic herbs that were not prescribed. However, because the NHI system provides a comprehensive coverage and because the copayment for prescriptions is consistently 50 NT$ (New Taiwan Dollar) (approximately equal to US $1.5), which is generally less than the cost of herbs that are sold in the markets of Taiwan, the likelihood that the subjects purchased a large amount of other phytoestrogen-containing herbs outside of the NHI database is low. Furthermore, because we had previously limited the cohort to women who did not receive prescription Chinese herbal products during the 12 years of observation, the likelihood that the subjects purchased herbs outside of the services of the NHI becomes even lower. Furthermore, when we limited our analysis to postmenopausal women (55 to 79 years of age) who did not require post-coitus emergency contraceptives or birth control pills, the results showed the same trend as that observed among women aged between 20 to 79 years. However, we are unable to rule out the possibility that the occurrence of breast cancer in postmenopausal women was attributed by the use of a contraceptive pill early in life. Third, we were unable to validate the actual ingested dose of the prescribed HT recorded in the database. A large mean cumulative dose indicates that the patient continued to receive the same prescription for a long period and implies that the patient actually consumed the prescribed medication. Even if the patient did not consume all of the prescribed HT pills, the present findings would only underestimate the effect of the consumed HT. Fourth, because the reimbursement data did not include the selection patterns of phytoestrogen-rich foods, the relative weight and reproductive history of the women, we were unable to control for this factor in the model construction. Because the present study included females from a random sampling cohort, we assumed that such confounding factors would not bias the results.

In summary, the present study found that current users of carefully prescribed E-alone or E+P in Taiwan have an increased risk for invasive breast cancer, especially elderly women. The overall risk seems to be less favourable for E+P in comparison to E-alone. Thus, we recommend that precautionary actions be taken toward the prescription of HT for any duration among postmenopausal women in Taiwan, and the current findings should be incorporated into the established guidelines and emerging risks and benefits of these agents.

## Materials and Methods

### Ethics statements

This study was initiated after approval by the review board of the Committee on Chinese Medicine and Pharmacy, Department of Health, Taiwan. All data were obtained from the NHI reimbursement database. The National Health Research Institutes of Taiwan anonymized and transformed the NHI reimbursement data as files suitable for research. Since the identification numbers and personal information of all individuals were not included in the above secondary files to protect the privacy of the individuals, the review board approved that written consents from patients were not required.

### Study population and data collection

In March of 1995, Taiwan established the National Health Insurance (NHI) programme, which routinely reimburses more than 96% of Taiwanese residents [27] for the cost of prescribed medicines since 1997. All reimbursement data for the NHI that are transformed and maintained by the National Health Research Institutes (NHRI) of Taiwan [28] contained detailed demographic data (including birth date and sex) and information regarding health-care services provided for each patient, including all of the payments for outpatient visits, hospitalisations, and prescriptions, and the residence of each patient. The data also contained that were documented for each outpatient visit or hospitalisation included up to five diagnoses that were coded according to the *International Classification of Diseases, Ninth Revision* (*ICD-9* ) [29], all of the drugs prescribed and their doses (i.e., conventional medicines, including generic and commercial brands of estrogen and progesterone drugs, and Chinese herbal medicines (CHM)), and the date of each prescription. To facilitate research, the NHRI has created a simple random sample of one million individuals from the 22 million insured populations (National Health Insurance Research Database) which cohort was further validated to be representative of the entire insured population [28].

**Figure 1:**
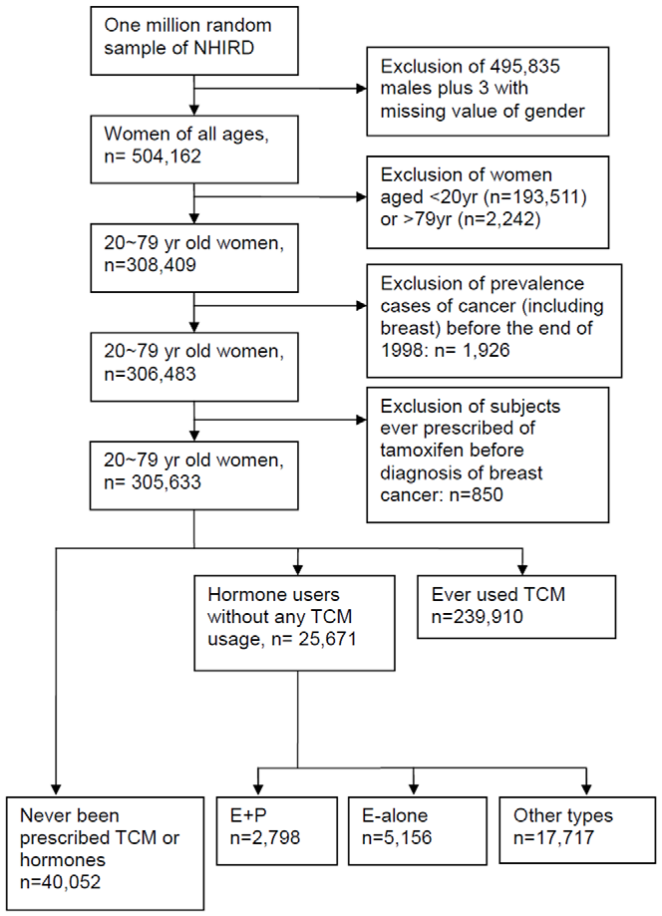
Algorithm of recruitment of subjects into the cohort from the one million random sample of the National Health Insurance Research Database (NHIRD) followed from 1997 to 2008 in Taiwan.

The selection of study subjects from the random sample of one million individuals was performed as follows (Figure 1). First, we excluded all of the male subjects (*n = *495,836) or those with missing information concerning gender (*n* = 3). Age was calculated by subtracting the subject's birthday from the 1^st^ day of July for each year. Second, subjects under 20 (*n = *193,511) or over 79 years of age (*n = *2,242) were excluded to limit the study sample to the main consumers of hormones in Taiwan. All of the patients with invasive breast cancer were included in the study, and the diagnosis was verified by the NHI catastrophic illnesses registry during 1998-2008. Because all patients who are confirmed to have a catastrophic illness are exempt from all copayments, the presence of invasive breast cancer must be validated by tissue pathology to qualify for the registry, and the case is further classified into one of the following categories: the nipple and areola (*ICD-9* codes 1740); central portion; upper-inner quadrant; lower-inner quadrant; upper-outer quadrant; lower-outer quadrant; axillary tail; unspecified (*ICD-9* code 1741, 1742, 1743, 1744, 1745, 1746, and 1749, respectively); other specified sites of the female breast (*ICD-9* code 1748). We excluded two years, 1997 and 1998, to avoid the inclusion of 1926 prevalent cases. To control potential confounding factors, we further excluded 850 subjects who had ever used tamoxifen prior to any diagnosis of breast cancer. Finally, 305,633 subjects qualified for inclusion in the cohort. 10,309 Chinese herbal products (CHPs) were licensed by Taiwan government and covered by the NHI. Several popular Chinese herbal products (CHP) contain small amounts of estrogen-like substances [10]-[14], [30] and are prescribed frequently in Taiwan, e.g., dong quai, ginseng , licorice, and ge gen, among others. Thus, we further excluded 239,910 subjects who had ever used CHP to make sure the results will not be biased by its inconclusive effects on breast cancer as shown in Table 4. Finally, we obtained a reference group of 40,052 subjects who had not used CHP or hormones during the study period and three exposed groups with demonstrated different types of hormone usage (5,156 prescribed E-alone, 2,798 E+P, and 17,717 others).

**Table 4:** Distribution frequencies of licensed and prescribed Chinese herbal products containing estrogen-like substance, 1999–2008*.

Chinese herbal products	No. of licensed	Major Corresponding (Metabolic) Active Substance(s) in Humans
Total licensed Chinese herbal products	10,309 (100)	
*Dong Quai*	2,492 (24.2)	estrogen-like effect
*Ginseng*	1,960 (19.0)	estrogen-like effect
*Licorice*	5,342 (51.8)	estrogen-like effect
Licensed Chinese herbal products containing estrogen-like substance	721 (7.0)	
*Psoraleae Fructus*	60 (0.6)	Genistein, daidzein and biochanin A, coumestrol
*Puerariae Radix*	505 (4.9)	Puerarin, daidzin, genistin, daidzein and genistein, coumestrol
*Sojae Semen Praeparatum*	74 (0.7)	Genistein, genistin, daidzein, daidzin, glycitein and glycitin, coumestrol
*Sophorae Flavescentis Radix*	75 (0.7)	Daidzein, genistein and formononetin
*Sophorae Immaturus Flos*	38 (0.4)	Genistin, genistein

*The table shows the distribution frequencies of licensed and prescribed Chinese herbal products (CHPs) that may contain estrogen-like substance.

### Exposure assessment for HT

A total of 25,671 women were prescribed at least one type of HT without an additional intake of any Chinese herbs during the study period from January 1, 1997 to December 31, 2008. All of the prescribed medications were covered under the NHI of Taiwan, and no drugs were dispensed at a pharmacy without a physician's prescription. The major indications for E-alone and E+P in women were contraception, dysmenorrhoea, hypogonadism (estrogen-deficient patients), menopausal hot flashes, and vaginal atrophy. The reimbursement database contained all the details regarding the prescribed conventional medicines, which included all of the types of HT and the commercial names of 14 types of estrogen-containing drugs and ten types of progestagen-containing drugs. The variables for HT usage that were included in the analyses were defined according to the specific proprietary preparation of HT used by the subjects during the 12-year study period. We categorised the types of preparations used as follows: E-alone; E+P; other preparations, including progesterone only and vaginal and other local treatments; combinations of the above preparation types.

### Statistical analyses

The incidence rate was summarised as the number of new invasive breast cancer patients/10^6^ person-years at risk. Using 40,052 non-hormone users as the reference group, we constructed multivariate Cox proportional hazard models to estimate the hazard ratios and their 95% confidence intervals (CI) for new occurrences of invasive breast cancer after adjusting for age. To minimise the potential confounding by indications for HT, we conducted another Cox regression using women who had ceased prescription hormone intake for 6 years or more as the reference group. An estimate with a 95% CI that did not contain the number 1 was considered statistically significant. All the above analyses were conducted using the SAS ver. 9.2 software package (SAS Institute, Cary, NC, USA).

## Supporting Information

Table S1 **Prevalence of HRT use and age-adjusted breast cancer incidence rates^a^ and 95% CI in Taiwanese and the United States.**
(DOC)

Table S2 **Age-specific breast cancer incidence rates^a^ in Taiwan, Japan, Hong Kong and American white women.**
(DOC)
