# Supplementary material for: Increased Risk for Invasive Breast Cancer Associated with Hormonal Therapy: A Nation-Wide Random Sample of 65,723 Women Followed from 1997 to 2008
Source: PLoS One. 2011 Oct 6;6(10):e25183. doi: 10.1371/journal.pone.0025183 (PMC3188542; doi:10.1371/journal.pone.0025183)
Supplement: Table S1 — Prevalence of HRT use and age-adjusted breast cancer incidence ratesa and 95% CI in Taiwanese and the United States. (DOC) [file pone.0025183.s001.doc]

Table S1. Prevalence of HRT use and age-adjusted breast cancer incidence rates^a^ and 95% CI in Taiwanese and the United States

| **Country**  Hormone therapies age, years | Prevalence of HRT use (year) |
| --- | --- |
| **United States** ^a^ |  |
| Hormone therapies 45+ years | - |
| E-alone | 15.6 % (1999-2006) |
| E+P | 13.4 % (1999-2006) |
| **Taiwan** ^b^ |  |
| Hormone therapies 20-79 years |  |
| E-alone | 7.8 % (1997-2008) |
| E+P | 4.3 % (1997-2008) |
| Mixed regimen | 15.2 % (1997-2008) |
| Hormone therapies 55-79 years |  |
| E-alone | 12.3 % (1997-2008) |
| E+P | 5.3 % (1997-2008) |
| Mixed regimen | 7.4 % (1997-2008) |

**^a^** Based on prevalence of HRT use among Kaiser Permanente Northwest members (i.e. women filling at least one prescription were considered HRT users).

^b^ Based on prevalence of HRT use among 65,723 Taiwanese women
