# Supplementary material for: Increased Risk for Invasive Breast Cancer Associated with Hormonal Therapy: A Nation-Wide Random Sample of 65,723 Women Followed from 1997 to 2008
Source: PLoS One. 2011 Oct 6;6(10):e25183. doi: 10.1371/journal.pone.0025183 (PMC3188542; doi:10.1371/journal.pone.0025183)
Supplement: Table S2 — Age-specific breast cancer incidence ratesa in Taiwan, Japan, Hong Kong and American white women. (DOC) [file pone.0025183.s002.doc]

Table S2. Age-specific breast cancer incidence rates^a^ in Taiwan, Japan, Hong Kong and American white women.

| Incidence | **Taiwan** ^b^ | **Japan** ^c^ | **Hong Kong** ^d^ | **American white** ^e^ |
| --- | --- | --- | --- | --- |
| years | 2000-2007 | 2000-2006 | 2000-2008 | 2000-2008 |
| Age |  |  |  |  |
| 20-24 | 1.7 | 0.8 | 1.0 | 1.4 |
| 25-29 | 7.9 | 5.4 | 6.1 | 7.6 |
| 30-34 | 24.7 | 19.4 | 19.4 | 25.5 |
| 35-39 | 57.1 | 48.3 | 47.7 | 59.4 |
| 40-44 | 95.7 | 98.4 | 95.8 | 121.3 |
| 45-49 | 130.9 | 148.6 | 133.4 | 191.6 |
| 50-54 | 126.8 | 134.5 | 130.6 | 239.7 |
| 55-59 | 130.1 | 127.7 | 139.0 | 303.9 |
| 60-64 | 122.2 | 132.3 | 128.6 | 376.3 |
| 65-69 | 104.2 | 119.3 | 117.8 | 433.6 |
| 70-74 | 87.8 | 111.6 | 119.6 | 447.6 |
| 75-79 | 76.6 | 106.7 | 130.7 | 473.7 |
| 80-84 | 69.2 | 94.3 | 152.8 | 448.3 |
| 85+ | 56.8 | 78.2 | 150.0 | 367.7 |

^a^ Rates are average annual per 100,000

^b^ Data from the Taiwan Cancer Registry (2000-2007)

^c^ Data from the Japan Cancer Registry (2000-2006)

^d^ Data from the Hong Kong Cancer Registry (2000-2008)

^e^ Rates are average annual per 100,000 for the following SEER areas (2000-2008): Atlanta, Detroit, Seattle/Puget Sound; and the states of California (registries for Los Angeles County, the Greater San Francisco Bay Area, and the rest of California), Connecticut, Hawaii, Iowa, Kentucky, Louisiana, New Jersey, New Mexico, and Utah
